# Metabolic Dysfunction-associated fatty liver disease and incident heart failure risk: the Kailuan cohort study

**DOI:** 10.1186/s13098-023-01102-0

**Published:** 2023-06-24

**Authors:** Zhihao Wei, Zhe Huang, Zongshuang Song, Wenliu Zhao, Dandan Zhao, Yizhen Tan, Shuohua Chen, Peng Yang, Yun Li, Shouling Wu

**Affiliations:** 1 School of Public Health, North China University of Science and Technology, Tangshan, 063210 China; 2 Department of Cardiology, Kailuan General Hospital, 57 Xinhua East Rd, Tangshan, 063000 China; 3 Department of Neurosurgery, Affiliated Hospital of North, China University of Science and Technology, Tangshan, 063000 China

**Keywords:** Heart failure, MAFLD, Cohort study, Type 2 diabetes

## Abstract

**Background:**

Recently, metabolic dysfunction-associated fatty liver disease (MAFLD) has been proposed to replace non-alcoholic fatty liver disease (NAFLD) to emphasize the pathogenic association between fatty liver disease and metabolic dysfunction. Studies have found that MAFLD independently increases the risk of myocardial infarction and stroke. But the relationship between MAFLD and heart failure (HF) is not fully understood.

**Objectives:**

This study aimed to explore the association between MAFLD and the risk of HF.

**Methods:**

The study included 98,685 participants without HF selected from the Kailuan cohort in 2006. All participants were divided into non-MAFLD group and MAFLD group according to MAFLD diagnostic criteria. After follow-up until December 31, 2020, the Cox regression analysis model was used to calculate the effect of MAFLD on the risk of HF.

**Results:**

During the median follow-up of 14.01 years,3260 cases of HF were defined, the HF incidence density of non-MAFLD group and MAFLD group was 2.19/1000pys and 3.29/1000pys, respectively. Compared with the non-MAFLD group, participants with MAFLD had an increased risk of HF (HR: 1.40, 95% CI: 1.30–1.50); in addition, an exacerbation of fatty liver disease was associated with an increased risk of HF in people with MAFLD. We also observed a higher risk of HF among the different metabolic dysfunction of MAFLD in people with both fatty liver disease and type 2 diabetes (HR, 1.95; 95% CI, 1.73–2.20).

**Conclusions:**

Our findings suggest that the risk of HF was significantly increased in participants with MAFLD, and an exacerbation of fatty liver disease was associated with an increased risk of HF in people with MAFLD. In addition, we should pay more attention to people with MAFLD with type 2 diabetes.

## Introduction

Heart Failure (HF) is the ultimate manifestation of various heart diseases [1]. Global Burden of Disease (GBD) data showed: by the end of 2017, the global number of HF cases reached 64.3 million, an increase of 91.9% from 1990, and the global age-standardized prevalence rate was 0.83% [2]. A Chinese chronic disease study found that the age-standardized incidence of HF in China in 2017 was 275/100,000 person-years, with a prevalence of 1.10%; the standardized prevalence of HF among people over 35 years old was 1.38%, an increase of about 50% from 2006; in addition, the average hospitalization cost of HF patients in China was as high as $4,406.8 in 2017 [3], it had brought huge economic burden to China’s health system and families. However, due to the lack of effective treatment for HF, finding modifiable risk factors and implementing effective interventions had become one of the main strategies for preventing HF [4–6].

Non-alcoholic fatty liver disease (NAFLD) was the most common liver disease in the world, with prevalence rates of 25.2% globally [7] and 29.6% in Asia [8]. Recently, metabolic dysfunction-associated fatty liver disease (MAFLD) has been proposed to replace NAFLD to emphasize the pathogenic association between fatty liver disease and metabolic dysfunction [9]. Studies have found that MAFLD independently increases the risk of myocardial infarction and stroke [10–12]; however, the relationship between MAFLD and HF has been less reported [10]. In addition, the above studies did not explore the relationship between fatty liver degree and HF in people with MAFLD. We investigated the association between MAFLD and HF in a Chinese population based on the Kailuan Cohort.

## Methods

### Study participants

The Kailuan Study was a prospective study conducted in the Kailuan Community of Tangshan, China. The detailed study design and procedures had been described in previous studies [13, 14]. Between June 2006 and October 2007, a total of 101,510 employees (81,110 males and 20,400 females) of the Kailuan Group were invited and agreed to participate in the Kailuan Study. Participants were followed up every 2 years. In this study, a total of 101,510 active and retired employees of Kailan Group who participated in physical examination in 2006 were included. Participants with previous history of malignant tumor (N = 377), participants with previous history of HF (N = 81), and participants with major data missing of MAFLD (N = 2367) were excluded. Finally, a total of 98,685 volunteers were enrolled.

### Data Collection

Information on demographic variables (e.g., history of use of antihypertensive, antiglycaemic or antilipidemic drugs) was collected through questionnaires; the design of epidemiological questionnaires and anthropometric methods were described in the published literature of this group [15]. After 5 min of rest in a chair, volunteers measured blood pressure in the left arm using the appropriate cuff size, averaging at least two readings of each systolic and diastolic blood pressure for further analysis. In addition, after 8 h of fasting, 5ml of elbow venous blood was drawn from the morning of the physical examination day for the detection of high density lipoprotein cholesterol (HDL-C), fasting blood glucose (FPG), triglycerides and high sensitive C-reactive protein (Hs-CRP), all of which were performed on Hitachi automated analyzers. Body mass index (BMI) = body mass (kg) / height ²(m²). Diabetes was defined as FPG ≥ 7.0 mmol/L, or self-reported use of antiglycaemic drugs, history of diabetes.

### Ascertainment of MAFLD

MAFLD was determined according to the recent consensus criteria [9]: MAFLD was defined as liver steatosis detected by ultrasonography in combination with one of the following three criteri: overweight/obesity (BMI ≥ 23.0 kg/m^2^), presence of type 2 diabetes, or evidence of metabolic dysregulation. In our study, metabolic dysregulation among thin/normal (BMI < 23.0 kg/m^2^) weight individuals with liver steatosis and who did not suffer from type 2 diabetes was determined by the presence of at least two of the following metabolic risk abnormalities:

1. Waist circumference ≥90 cm in men and 80 cm in women
2. Blood pressure ≥ 130/85 mmHg or specific drug treatment
3. TG ≥ 1.70 mmol/L or specific drug treatment
4. HDL-C <1.0 mmol/L for men and < 1.3 mmol/L for women, or specific drug treatment
5. Prediabetes (FPG levels of 5.6 to 6.9 mmol/L)
6. Plasma high-sensitivity C-reactive protein level > 2 mg/L; homeostasis model assessment-insulin resistance score was unavailable in our study.

The severity of steatosis was differentiated by ultrasonography: mild (diffuse increase in fine echoes in liver parenchyma), moderate (diffuse increase in fine echoes with impaired visualization of the intrahepatic vessel borders and diaphragm), and severe (diffuse increase in fine echoes with non-visualization of the intrahepatic vessel borders and diaphragm) [16]. Abdominal ultrasonography was routinely performed by experienced radiologists using a high-resolution B-mode topographical ultrasound system with a 3.5 MHz probe (ACUSON X300, Siemens, Germany) in the Kailuan study.

### Follow-up and assessment of incident HF

Starting with the 2006 physical examination and last follow-up on December 31, 2020, the outcome event of the study was the first occurrence of HF. The definition of HF was revised according to the diagnostic criteria of HF in Chinese Guidelines for the Diagnosis and Treatment of HF 2018. The diagnostic criteria included clinical manifestations, laboratory tests, and imageology. General cardiologists reviewed the medical records of patients and proved the diagnosis of HF according to the following criteria: (1) the clinical features of HF, such as difficulty in breathing, weakness, and fluid retention (e.g., ascites, pleural effusion, pedal edema, and increased jugular venous pressure), diagnosed with the New York Heart Association (NYHA) cardiac function class ≥ II or Killip cardiac function class ≥ II; (2) Doppler echocardiography showed the left ventricular ejection fraction (LVEF) ≤ 50%; (3) increased level of N-terminal Pro-B-type natriuretic peptide (NT-proBNP). The diagnosis of HF was confirmed by the presence of (1) and any of (2), (3).

### Statistical analyses

All analyses were performed using SAS, version 9.4 (SAS Institute, Inc, Cary, NC). Two-sided values of P < 0.05 were regarded as significant. Continuous variables with normal distribution were expressed as means ± SDs and compared using Student T test, while those with skewed distribution were expressed as medians and interquartile range and compared by Kruskal-Wallis test. Categorical variables were shown in proportions and compared by Pearson’s Chi-Square test. The Cox regression model was used to predict the risk of HF in MAFLD and its metabolic disorder types, and the degree of fatty liver degeneration. The cumulative incidence of HF in different groups was calculated by the Kaplan-Meier method, and the Log-rank test was used for comparison between groups. With HF as the dependent variable and MAFLD as the independent variable, stratified analysis by age. To verify the robustness of the results, we repeated the primary analysis, excluding participants who developed myocardial infarction during follow-up for sensitivity analyses. The model was adjusted for age, sex, education level, smoking, alcohol consumption, physical activity, use of antihypertensive drugs, use of antiglycemic medications, and use of antilipidemic medications.

## **Results**

### Participant characteristics

A total of 98,685 participants (mean age 51.88 ± 12.64 years) were enrolled, of whom 78,893 (79.94%) were men. In this study, the number of participants in the Non-MAFLD group and MAFLD group was 67,930 and 30,755, respectively; of these, 4290 participants dropped out during follow-up. As shown in Table 1, compared with the non-MAFLD group, the values of TG, BMI, FBG, Hs-CRP in the MAFLD group were higher; in addition, hypertension and diabetes also had a high proportion in the MAFLD group (all p < 0.01).

**Table 1:** Baseline Clinical Characteristics According to MAFLD Status(N = 98,685)

	Total	Non-MAFLD	MAFLD	p-value
Participants (n)	98,685	67,930	30,755	
Age,year	51.88 ± 12.64	51.48 ± 13.12	52.75 ± 11.49	< 0.01
Male, N(%)	78,893(79.94)	53,697(79.05)	25,196(81.92)	< 0.01
Mean follow-up time,year	14.01(13.63–14.19)	14.00(13.63–14.19)	14.01(13.61–14.20)	0.24
SBP, mmHg	131.09 ± 21.05	128.24 ± 20.49	137.36 ± 20.92	< 0.01
DBP, mmHg	83.51 ± 11.79	81.78 ± 11.37	87.35 ± 11.79	< 0.01
TG, mmol/L	1.27(0.90–1.93)	1.12(0.80–1.60)	1.77(1.23–2.67)	< 0.01
BMI, Kg/m^2^	25.05 ± 3.49	23.94 ± 3.05	27.50 ± 3.14	< 0.01
FBG, mmol/L	5.11(4.66–5.71)	5.04(4.60–5.56)	5.36(4.81–6.16)	< 0.01
HDL-C, mmol/L	1.50(1.28–1.77)	1.51(1.29–1.77)	1.48(1.26–1.74)	< 0.01
WC,cm				< 0.01
female	83.06 ± 10.73	80.24 ± 10.01	90.27 ± 9.01	
males	83.06 ± 9.57	85.67 ± 9.02	93.24 ± 8.62	
Hs-CRP,mg/L	0.80(0.30–2.20)	0.69(0.26–1.82)	1.20(0.50–3.01)	< 0.01
Current smoker, N (%)	34,018(34.47)	23,311(34.32)	10,707(34.81)	0.13
Current drinker, N (%)	40,818(41.36)	27,480(40.45)	13,338(43.37)	< 0.01
Physical activity, N(%)	90,075(91.28)	61,874(91.08)	28,201(91.70)	< 0.01
Education level, N(%)				< 0.01
≤junior high school	78,655(79.70)	53,970(79.45)	24,685(80.26)	
≥senior high school	20,030(20.30)	13,960(20.55)	6070(19.74)	
Salt level, g/d				< 0.01
< 6	9163(9.29)	6472(9.53)	2691(8.75)	
6–10	78,843(79.89)	54,436(80.14)	24,407(79.36)	
> 10	10,679(10.82)	7022(10.34)	3657(11.89)	
Antihypertensive treatment, N(%)	11,029(11.18)	5762(8.48)	5267(17.13)	< 0.01
Antidiabetic treatment, N (%)	2420(2.45)	1221(1.80)	1199(3.90)	< 0.01
Lipid-lowering treatment, N (%)	925(0.94)	431(0.63)	494(1.61)	< 0.01
hypertension	37,128(37.62)	21,380(31.47)	15,748(51.20)	< 0.01
diabetes	9336(9.46)	4278(6.30)	5058(16.45)	< 0.01

Data were present as n (%), mean ± SD, or median (P25, P75) according to variable category. Pearson’s chi-square test, Student T test, or Kruskal-Wallis test was used to compare differences between groups properly

Abbreviations: BMI, body mass index; FBG, fasting blood glucose; SBP, systolic blood pressure; DBP, diastolic blood pressure; WC, waist circumference; HDL-C, high-density lipoprotein; TG, triglyceride

### Association between MAFLD and HF

**Fig. 1:**
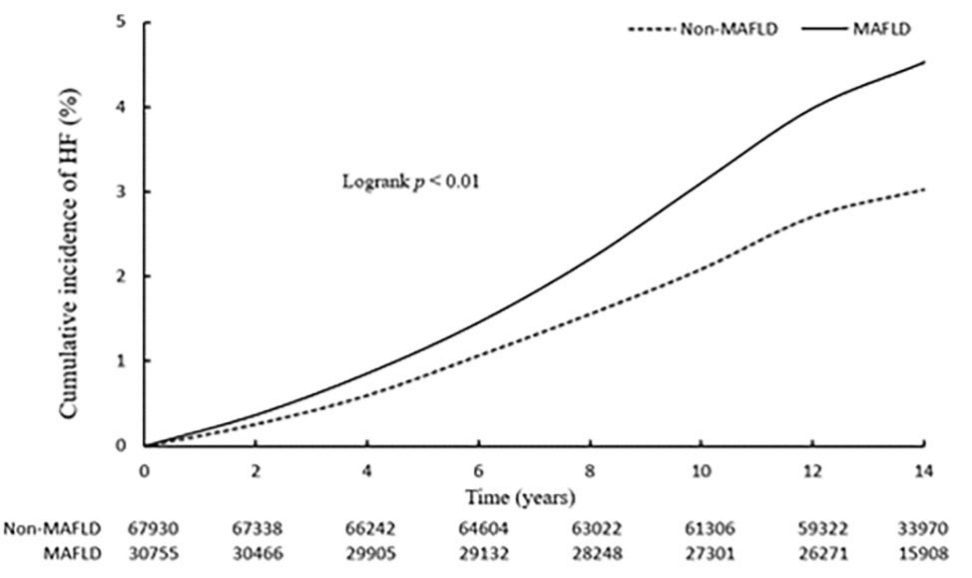
Cumulative incidence of heart failure at different MAFLD levels

In the study with a median follow-up of 14.01 years, 3260 cases of HF were defined, and the incidence rate (3.29/1000pys) was higher in the MAFLD group than that in the non-MAFLD group (2.19/1000pys). After adjusting for covariates, the risk of HF remained significant in the MAFLD group (HR: 1.40; 95% CI: 1.30 to 1.50) (Table 2) (Fig. 1).

**Table 2:** Hazard Ratios for HF According to MAFLD Status and its subgroups

	Case/Participants	Incidence (/1, 000 person years)	HR (95%CI)		
			Model 1	Model 2	Model 3
**Total**					
Non-MAFLD	1945/67,930	2.19(2.09,2.28)	reference	reference	reference
MAFLD	1315/30,755	3.29(3.12,3.47)	1.51(1.40,1.62)	1.52(1.42,1.63)	1.40(1.30,1.50)
**Group by the degree of fatty liver**					
Non-MAFLD	1945/67,930	2.19(2.09,2.28)	reference	reference	reference
Mild MAFLD	809/20,035	3.10(2.90,3.32)	1.41(1.30,1.53)	1.43(1.31,1.55)	1.33(1.23,1.45)
Moderate MAFLD	412/8775	3.62(3.29,3.99)	1.66(1.50,1.85)	1.68(1.50,1.86)	1.51(1.35,1.68)
Severe MAFLD	94/1945	3.77(3.08,4.61)	1.82(1.50,2.24)	1.85(1.50,2.27)	1.57(1.28,1.94)
**Group by type of metabolic disorder**					
Non-MAFLD	1945/67,930	2.19(2.09,2.28)	reference	reference	reference
MAFLD1^*^	895/24,590	2.77(2.59,2.96)	1.31(1.21,1.42)	1.33(1.22,1.43)	1.27(1.17,1.38)
MAFLD2^*^	37/1107	2.64(1.91,3.64)	1.09(0.79,1.51)	1.10(0.79,1.52)	1.12(0.81,1.55)
MAFLD3^*^	383/5058	6.14(5.56,6.79)	2.42(2.17,2.70)	2.43(2.18,2.71)	1.95(1.73,2.20)

*MAFLD1: BMI ≥ 23 kg/m2 without diabetes

MAFLD2: BMI < 23 kg/m2 with at least two metabolic abnormalities but not diabetes

MAFLD3: type 2 diabetes mellitus

Model 1: adjusted for age, gender;

Model 2: adjusted for all the variables in model 1 and Smoking, Drinking, Education level, Salt status and Physical activity;

Model 3: adjusted for all the variables in model 2 and Antihypertensive treatment, Antidiabetic treatment and Lipid-lowering treatment

### Subgroup analysis of the effect of MAFLD on HF

The MAFLD group was classified according to the degree of fatty liver and divided into the mild fatty liver group (n = 20,035), moderate fatty liver group (n = 8775) or severe fatty liver group (n = 1945). Severe fatty liver (HR: 1.57; 95% CI: 1.28–1.94) and moderate fatty liver (HR: 1.51; 95% CI: 1.35–1.68) significantly increased the risk of HF compared with non-MAFLD, and mild fatty liver disease (HR: 1.33; 95% CI: 1.23–1.45) also increased the risk of HF disease (Table 2).

We further divided MAFLD into three subgroups based on metabolic dysfunction, MAFLD1 (BMI ≥ 23 kg/m2 without diabetes; n = 24,590), MAFLD2 (BMI < 23 kg/m2 with at least two metabolic abnormalities but not diabetes; n = 1,107) and MAFLD3 (type 2 diabetes mellitus; n = 5,058). Model 3 showed that both the MAFLD3 (HR, 1.95; 95% CI, 1.73–2.20) and MAFLD1 (HR, 1.27; 95% CI, 1.17–1.38) significantly increased the risk of HF disease compared with the non-MAFLD group; However, the MAFLD2 failed to increase the risk of HF disease (HR, 1.12; 95% CI, 0.81–1.55). The results showed that patients with diabetes in the MAFLD population were at higher risk of developing HF. (Table 2).

### **Association between MAFLD and HF by age**

There was an interaction between MAFLD and age (P < 0.01), and we further analyzed the effect of MAFLD on the risk of HF through age stratification. The association between MAFLD and HF was found to be significant in people under 45 years of age (HR, 1.82; 95% CI, 1.37, 2.42); MAFLD remained at significant risk of HF in people aged 45 to 65 years (HR, 1.50; 95% CI, 1.36, 1.65). However, the association between MAFLD and HF was low in people > 65 years of age (HR, 1.13; 95% CI, 1.01, 1.28) (Table 3).

**Table 3:** Hazard Ratios for HF According to MAFLD Status, and by age

	Case/Participants	Incidence (/1, 000 person years)	HR (95%CI)		
			Model 1	Model 2	Model 3
**Age group**					
**age < 45 years**					
Non-MAFLD	108/20,974	0.37(0.31,0.45)	reference	reference	reference
MAFLD	97/7574	0.93(0.77,1.40)	1.98(1.50,2.62)	1.99(1.51,2.63)	1.82(1.37,2.42)
**45 ≤ age < 65 years**					
Non-MAFLD	950/36,247	1.97(1.85,2.10)	reference	reference	reference
MAFLD	789/18,734	3.20(2.98,3.43)	1.63(1.48,1.80)	1.65(1.50,1.81)	1.50(1.36,1.65)
**age ≤ 65 years**					
Non-MAFLD	887/10,709	7.54(7.06,8.05)	reference	reference	reference
MAFLD	429/4447	8.80(8.00,9.67)	1.19(1.06,1.34)	1.20(1.07,1.35)	1.13(1.01,1.28)
Sensitivity 1*					
Non-MAFLD	1811/67,796	2.04(1.95,2.13)	reference	reference	reference
MAFLD	1216/30,656	3.05(2.88,3.23)	1.50(1.39,1.61)	1.51(1.40,1.62)	1.39(1.29,1.49)
Sensitivity 2*					
Non-MAFLD	1768/67,753	1.99(1.90,2.08)	reference	reference	reference
MAFLD	1202/30,642	3.01(2.84,3.18)	1.51(1.41,1.63)	1.53(1.42,1.64)	1.41(1.31,1.52)

* Sensitivity 1: excluding 233, myocardial infarction occurred during follow-up

Sensitivity 2: excluding 290, heart failure developed within two years

Model 1: adjusted for age, gender;

Model 2: adjusted for all the variables in model 1 and Smoking, Drinking, Education level, Salt status and Physical activity;

Model 3: adjusted for all the variables in model 2 and Antihypertensive treatment, Antidiabetic treatment and Lipid-lowering treatment

### Sensitivity analyses

Myocardial infarction was a risk factor for HF, and after myocardial infarction, scar tissue in part of the heart can replace normal myocardial tissue and affect the heart’s pumping function, which in turn leads to the occurrence of HF; in order to prevent the impact of myocardial infarction on the outcome, we excluded myocardial infarction events that occurred during follow-up (N = 233), and the results were similar to the primary results (HR, 1.39; 95% CI 1.29–1.49) (Table 3). In addition, MAFLD can affect HF, and HF may also worsen the severity of MAFLD. To reduce its reverse causation, we removed people with HF that occurred within two years for sensitivity analysis, and the results did not change significantly.

## Discussion

In this large community-based prospective study, we found that MAFLD was a risk factor for new onset HF independent of traditional risk factors; in addition, an exacerbation of fatty liver disease was associated with an increased risk of HF in people with MAFLD.

A Korean study found a 1.67-fold increased risk of HF in people with MAFLD [17]. However, the above studies lacked data on antihypertensive, hypoglycemic, and lipid-lowering drugs. After adjusting antihypertensive, hypoglycemic and lipid-lowering drugs, our results showed a 1.40-fold increased risk of HF in the MAFLD group compared to the non-MAFLD group. Our findings were consistent with those in Korea and found that MAFLD increases the risk of HF.

In addition, our study found that the worsening of fatty liver disease is associated with an increased risk of HF in people with MAFLD. The results were similar to those found in the NAFLD population, Jiyun Park et al. found that the degree of fatty liver disease in NAFLD people characterized by fatty liver index is associated with an increased risk of CVD [18–21], NAFLD participants with a fatty liver index greater than 60 had a higher risk of HF [21]. However, the above study only qualified the degree of fatty liver through fatty liver index, and still lacked relevant imaging data. In this study, ultrasound data were used instead of fatty liver index to characterize the severity of liver fat. Compared with histology, ultrasound can more accurately detect moderate and severe fatty liver [22]. Moderate to severe liver fat may aggravate insulin resistance in the liver; As the target organ and the starting organ of insulin resistance [23], the liver secreted more pro-inflammatory factors, affected myocardial metabolism, caused microcirculation disorders, and thus led to structural and functional disorders of the heart [24]. In addition, studies had found that patients with moderate to severe fat have a higher risk of left ventricular diastolic dysfunction and cardiac remodeling [25], and the severity of fatty liver was significantly correlated with changes in cardiac structure. These studies supported our findings to some extent.

In addition, we observed a higher risk of HF among the different metabolic dysfunction of MAFLD in people with both fatty liver disease and type 2 diabetes. On the one hand, diabetes mellitus was an independent risk factor that increases the risk of HF. On the other hand, studies had found a pathological association between diabetes and fatty liver, and type 2 diabetes and insulin resistance can promote the development of advanced liver fibrosis in patients with NAFLD or MAFLD [26, 27]; Similarly, fat accumulation in the liver can lead to hepatic insulin resistance and increase glucose production in the liver, thereby increasing the risk of systemic insulin resistance [28] and CVD [29]. However, previous studies on the association between MAFLD and heart failure had not been stratified by age. Age differences in increased risk of heart failure were observed in our population, with MAFLD associated with a higher risk of heart failure in the lower age group (< 45 years); This finding may be related to the effect of hypertension, metabolic syndrome, and diabetes on cardiovascular disease, which are higher in the MAFLD population than in the non-MAFLD population. One study found that participants diagnosed with metabolic syndrome at age < 45 had a higher risk of subsequent CVD, with a HR of 1.84 [30]; Similarly, subjects with hypertension and type 2 diabetes diagnosed at < 45 years had a higher relative risk of subsequent CVD, with AHRs of 1.84 and 3.21, respectively. The study found that the risk of CVD varied among age groups with type 2 diabetes, metabolic syndrome, and hypertension, and the association was more pronounced in subjects with young onset [31–33]. In addition, post-myocardial infarction is a risk factor for the occurrence of HF. Scar tissue of part of the heart can replace normal myocardial tissue after myocardial infarction and affect the pumping function of the heart, thus leading to the occurrence of HF. In order to prevent the influence of myocardial infarction on the outcome, myocardial infarction events occurred during follow-up were excluded, and the association did not change significantly.

The diagnosis of MAFLD includes cardiometabolic risks such as obesity /overweight, type 2 diabetes, and metabolic disorders. Obesity/overweight [34, 35], Type 2 diabetes [36] were associated with an increased risk of HF. In addition, the interaction between MAFLD and insulin resistance increases the levels of very low-density lipoprotein particles and triglycerides, leading to insulin receptor dysfunction, which mobilizes liver adipose tissue for transport to peripheral tissue and increases the risk of HF [37, 38]. In addition to insulin resistance, the proinflammatory state and increased oxidative stress in patients with MAFLD can lead to endothelial dysfunction and induce vascular inflammation, which contributes to the formation of atherosclerotic plaques and the development of HF [39].

This study not only provided a new clinical basis for the prevention of heart failure, but also provided a new theoretical basis for understanding the harm of MAFLD. Therefore, while paying attention to the harm of MAFLD disease, further attention should be paid to the harm caused by different degrees of fatty liver and types of metabolic disorders in MAFLD. For patients with MAFLD, the risk of heart failure should be reduced as much as possible and the quality of life should be improved by improving diet and lifestyle, controlling blood sugar, body weight, metabolic disorders and other types of metabolic disorders. For the risk population without MAFLD, the occurrence and development of fatty liver and metabolic disorders should be prevented in advance.

There were still limitations to the study. First, the lack of data on insulin resistance in our cohort and the inability to evaluate homeostasis models might have led to the misclassification of some participants with MAFLD, leading to an underestimation of the association between MAFLD and heart failure. Second, our study only collected heart failure (HFrEF) with reduced ejection fraction, and the results may not apply to other conditions with HF. Finally, the study subjects were the Kailuan Group population in northern China, which was not enough to represent all the population, and this result needs to be verified in other populations.

## Conclusion

In summary, MAFLD independently increased the risk of HF during a median follow-up period of 14.01 years. However, HF events depend primarily on the type of accompanying metabolic dysfunction. Based on the types of metabolic dysfunction included in the current definition of MAFLD, further research is necessary to refine the definition of MAFLD in order to improve the predictability of HF risk in different populations, given the heterogeneity of clinical outcomes of MAFLD.

## Data Availability

The data that support the findings of this study are available from [third party name] but restrictions apply to the availability of these data, which were used under license. for the current study, and so are not publicly available. Data are however available. from the corresponding author upon reasonable request and with permission of the corresponding author.

## Abbreviations

BMI: Body Mass Index
CI: Confidence Interval
DBP: Diastolic Blood Pressure
FBG: Fasting Blood Glucose
HDL: High Density Cholesterol
HR: Hazard Ratio
SBP: Systolic Blood Pressure
WC: Waist Circumference
TG: Triglyceride
Hs-CRP: High Sensitive C-reactive Protein
MAFLD: Metabolic Dysfunction-Associated Fatty Liver Disease
NAFLD: Non-alcoholic fatty liver disease
